# Centipeda minima active components and mechanisms in lung cancer

**DOI:** 10.1186/s12906-023-03915-y

**Published:** 2023-03-23

**Authors:** Cuiyun Gao, Huafeng Pan, Fengjun Ma, Ze Zhang, Zedan Zhao, Jialing Song, Wei Li, Xiangzhen Fan

**Affiliations:** 1 Department of Rehabilitation Medicine, Binzhou Medical University Hospital, Binzhou, Shandong China; 2 School of Rehabilitation Medicine, Binzhou Medical University, Yantai, Shandong China; 3 Science and Technology Innovation Center, Guangzhou University of Chinese Medicine, Guangzhou, Guangdong China; 4 Shandong University of Traditional Chinese Medicine, Jinan, Shandong China

**Keywords:** Centipeda minima, Lung cancer, Network pharmacology, Molecular docking, Experimental validation, Molecular mechanism

## Abstract

**Background:**

Traditional Chinese medicine (TCM) has been extensively used for neoplasm treatment and has provided many promising therapeutic candidates. We previously found that Centipeda minima (*C. minima*), a Chinese medicinal herb, showed anti-cancer effects in lung cancer. However, the active components and underlying mechanisms remain unclear. In this study, we used network pharmacology to evaluate *C. minima* active compounds and molecular mechanisms in lung cancer.

**Methods:**

We screened the TCMSP database for bioactive compounds and their corresponding potential targets. Lung cancer-associated targets were collected from Genecards, OMIM, and Drugbank databases. We then established a drug-ingredients-gene symbols-disease (D-I-G-D) network and a protein–protein interaction (PPI) network using Cytoscape software, and we performed Gene Ontology (GO) and Kyoto Encyclopedia of Genes and Genomes (KEGG) enrichment analyses using R software. To verify the network pharmacology results, we then performed survival analysis, molecular docking analysis, as well as in vitro and in vivo experiments.

**Results:**

We identified a total of 21 *C. minima* bioactive compounds and 179 corresponding targets. We screened 804 targets related to lung cancer, 60 of which overlapped with *C. minima.* The top three candidate ingredients identified by D-I-G-D network analysis were quercetin, nobiletin, and beta-sitosterol. PPI network and core target analyses suggested that TP53, AKT1, and MYC are potential therapeutic targets. Moreover, molecular docking analysis confirmed that quercetin, nobiletin, and beta-sitosterol, combined well with TP53, AKT1, and MYC respectively. In vitro experiments verified that quercetin induced non-small cell lung cancer (NSCLC) cell death in a dose-dependent manner. GO and KEGG analyses found 1771 enriched GO terms and 144 enriched KEGG pathways, including a variety of cancer related pathways, the IL-17 signaling pathway, the platinum drug resistance pathway, and apoptosis pathways. Our in vivo experimental results confirmed that a *C. minima* ethanol extract (ECM) enhanced cisplatin (CDDP) induced cell apoptosis in NSCLC xenografts.

**Conclusions:**

This study revealed the key *C. minima* active ingredients and molecular mechanisms in the treatment of lung cancer, providing a molecular basis for further *C. minima* therapeutic investigation.

**Supplementary Information:**

The online version contains supplementary material available at 10.1186/s12906-023-03915-y.

## Introduction

Lung cancer is one of the most common malignancies and the leading cause of all cancer-related deaths worldwide [1, 2]. Non-small cell lung cancer (NSCLC) represents approximately 85% of lung cancer cases. Current treatments for NSCLC mainly include surgery, radiotherapy, chemotherapy, targeted therapy, or some combination of these [3, 4]; however, the therapeutic efficacy of available treatments is not yet satisfactory. The majority of NSCLC patients have advanced disease at diagnosis and are not candidates for surgery [5, 6] and chemotherapy and radiotherapy effectiveness is frequently limited due to the onset of drug resistance and adverse effects. Thus, alternative therapeutic strategies are urgently needed for NSCLC treatment.

Many natural products and derivatives, such as Vinca alkaloids, taxanes, and camptothecins, have become part of the standard neoplasm chemotherapy repertoire [7]. Traditional Chinese medicine (TCM) has been used in cancer treatment for thousands of years, providing a rich basis for anti-carcinogen discovery and development. TCM products have been shown to exert anti-cancer effects through multiple signaling pathways and molecular targets [8]. Centipeda minima (L.)A.Br.et Aschers (*C. minima*), also called Ebushicao in TCM, belongs to the daisy family Asteraceae [9], is native to the tropical regions, and is famous for its extraordinary efficacy in treating nasal allergy, whooping cough, asthma, and malaria in TCM [10]. Phytochemical studies revealed that *C. minima* chemical constituents mainly include terpenoids, flavonoids, sterols, saponins, monophenols, fatty acids, and amides [11]. Crude extracts and purified compounds from *C. minima* have been shown to exert promising anti-cancer effects in a variety of human malignancies such as lung cancer, multiple myelomanasopharyngeal carcinoma, breast cancer, and colon cancer [12–15]. Nevertheless, the *C. minima* ingredients with anti-cancer activities are poorly characterized. Our previous studies found that a *C. minima* ethanol extract (ECM) combined with cisplatin (CDDP) had synergistic anti-cancer effects both in vitro and in vivo [16]; however, the underlying molecular mechanisms remain ill-defined.

**Fig. 1:**
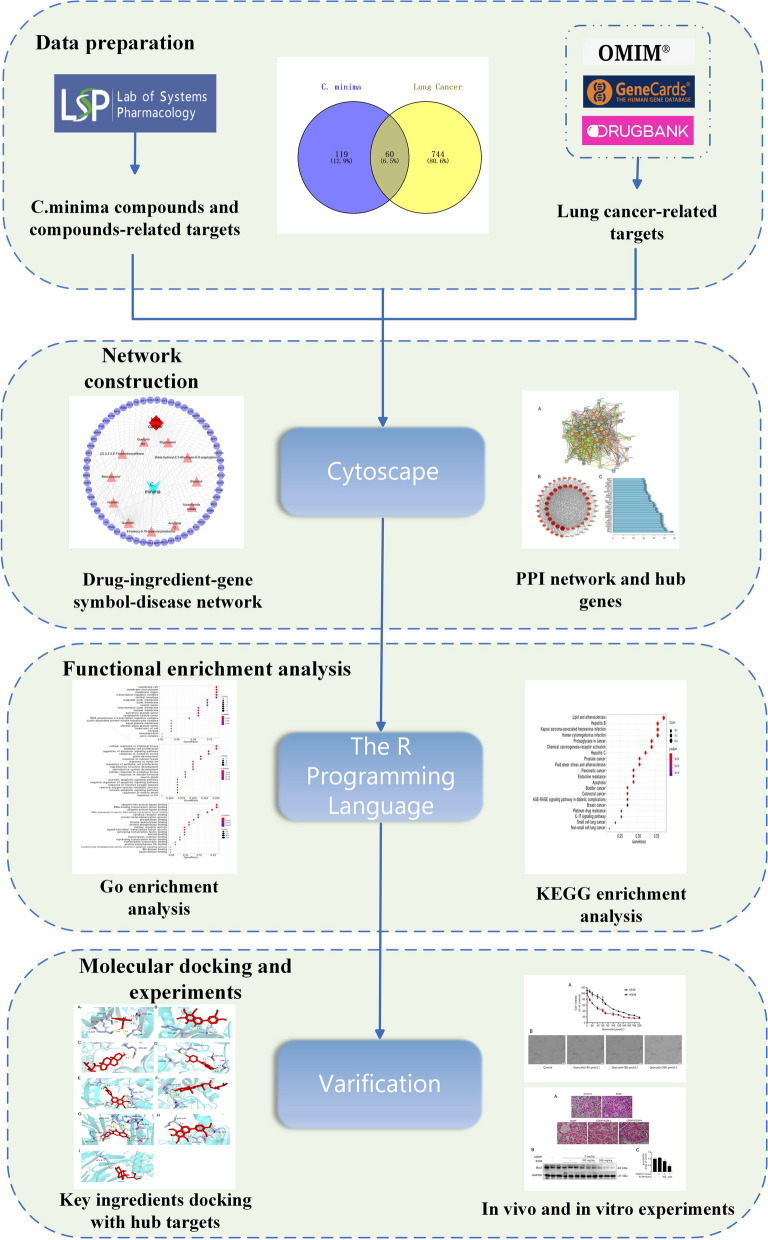
Schematic diagram summarizing how mechanisms associated with *C. minima* in lung cancer were identified using network pharmacology, molecular docking, and experimental validation

Here we applied network pharmacology and molecular docking to explore *C. minima* active ingredients and mechanisms against NSCLC. Network pharmacology, based on the general ideas of systems biology, is considered to be an efficient method for identifying drug mechanisms of action, and molecular docking is a powerful tool used in structural molecular biology and in computer-aided drug design [17]. Furthermore, we performed in vitro and in vivo experiments to verify the obtained results (Fig. 1).

## Materials and methods

### Screening for *C. minima* active components and targets

The Traditional Chinese Medicine Systems Pharmacy Database and Analysis Platform (TCMSP, http://lsp.nwu.edu.cn/tcmsp.php) is a unique systems pharmacology platform for Chinese herbal medicines that captures relationships among drugs, targets, and diseases. All ingredients were annotated using the Mol ID which is a unique numbering system for the TCMSP database. To identify *C. minima* compounds that may influence NSCLC, we preliminarily screened compounds collected from the TCMSP database using oral bioavailability (OB) and drug-likeness (DL) as parameters. OB is an important pharmacokinetic parameter used to measure a drug’s ADME (absorption, distribution, metabolism, excretion) in vivo. DL refers to the structural similarity between herbal ingredients and known drugs, and it plays a major role in new drug development. In this study we selected compounds with OB ≥ 30% and DL ≥ 0.18 as candidate active ingredients for further analysis. We searched for active compound standard names in the PubChem database (https://pubchem.ncbi.nlm.nih.gov/). Chemical constituents’ protein targets were obtained from the TCMSP database with OB ≥ 30% and DL ≥ 0.18 as screening conditions. Chemical ingredient and target databases were constructed using the data extracted from the TCMSP database.

### Target prediction

Lung cancer related targets were obtained from the Online Mendelian Inheritance in Man database (OMIM, http://omim.org/), Genecards database (https://www.genecards.org/), and Drugbank database (http://www.drugbank.ca/) using “lung cancer” as the keyword. The chemical constituents’ targets collected above were subsequently mapped to the lung cancer related targets, and the candidate targets related to both *C. minima* and lung cancer were obtained using VENNY2.1 (https://bioinfogp.cnb.csic.es/ tools/ venny/). All targets were finally identified and normalized using the UniProt database (https://www.uniprot.org/).

### Drug-ingredients-gene symbols-disease (D-I-G-D) network construction

Cytoscape [18] is an open source software platform that visualizes complex networks by integrating any type of attribute information. We imported the identified candidate targets, bioactive compounds, diseases, and herbs into Cytoscape 3.7.2 to construct a D-I-G-D network. In this network, nodes indicate drugs, active ingredients, targets, and diseases, and the edges indicate the interactions between them. The network analyzer Cytoscape plug-in was applied to obtain network topology parameters, such as nodes, degrees, and edge betweenness.

### Protein–protein interaction (PPI) network construction

To obtain protein–protein interaction relationships, candidate target gene data were imported into the STRING database (https://string-db.org/) [19] with “Homo sapiens” as the selected species and the minimum required interaction score limited to a medium confidence score of 0.400. The results were downloaded in TSV format and imported into Cytoscape 3.7.2 to visualize the PPI network interactions. The CytoHubba Cytoscape plugin was used to identify hub genes in the PPI network.

### Bioinformatic annotation

We input candidate targets into R4.0.5 software to perform GO and KEGG enrichment analyses. *P*-value < 0.05 and Q-value < 0.05 were considered statistically significant. The GO enrichment analysis included cellular components (CC), biological processes (BP), and molecular functions (MF). The top twenty significant terms for each category are presented, and bubble diagrams were drawn using R4.0.5 software. KEGG enrichment analysis clarified *C. minima* potential mechanisms against lung cancer, and we constructed bubble charts depicting the top 20 significant pathways [20].

### NSCLC gene expression dataset analysis

We used the Kaplan–Meier plotter (http://www.kmplot.com) to determine the correlation between hub gene expression and NSCLC patient prognosis.

### Component-target molecular docking

Molecular docking is a method used to study intermolecular interactions and predict their binding modes and affinity [21]. Here, we used it to confirm interactions between *C. minima* core compounds and hub targets in treating lung cancer and to verify the network pharmacology predictions’ accuracy. The specific process was as follows:

2.7.1 Small ligand molecule file preparation: Three-dimensional (3D) files for *C. minima* were downloaded in SDF format from the PubChem database and converted to PDB format using OpenBabel. They were saved as PDBQT ligand files using AutoDock Tools 1.5.6 software.

2.7.2 Macromolecular receptor file preparation: Receptor 3D structures were obtained from the Protein Data Bank (PDB, http://www.rcsb.org/, in PDB file format). Pymol 2.5.2 software was then used to extract the target proteins’ original ligand conformation by removing water molecules, co-crystallized ligand, and ions. Subsequently, AutoDock tools 1.5.6 was used to add polar hydrogen and Gasteiger charge to the processed receptors’ structures. The structures were then saved as PDBQT protein receptor files.

2.7.3 Defining docking parameters: The receptor and ligand PDBQT structures were imported into Autodock Tools 1.5.6 and the molecular docking range was defined. The docking center was set on the macromolecule, and the center coordinate (center x/y/z) and box size (size x/y/z) parameters were set to keep the protein completely covered by the docking box.

2.7.4 Docking and visualization: Autodock Vina 1.1.2 was used to conduct molecular docking and calculate docking affinity. The molecular docking results analysis refers to the binding energy (ΔGbind). A binding energy less than -5 kJ∙mol^−1^ indicates that the target has certain binding activity with the compound [22]. The lower the binding energy, the better the docking effect. Finally, the compound and protein docking results were analyzed and visualized using Pymol 2.5.2.

### Experimental validation

#### Chemicals and reagents

CDDP was purchased from Sigma Aldrich (St. Louis, MO, USA). Primary antibodies against Bcl-2 and GAPDH and all secondary antibodies were purchased from Cell Signaling Technology (Beverly, MA, USA). High purity (98.02%) quercetin was obtained from MedChemExpress (Monmouth Junction, NJ, USA).

#### Cell culture

A549 and H1299 cells, two commonly used NSCLC cell lines, were purchased from the American Type Culture Collection (ATCC, Manassas, VA, USA). A549 cells were cultured in Dulbecco’s Modified Eagle Medium (DMEM) supplemented with 10% fetal bovine serum (FBS). H1299 cells were maintained in RPMI-1640 medium containing 10% FBS. All reagents listed above were purchased from Gibco (CA, USA). Cells were cultured in a humidified incubator with 5% CO_2_ at 37 °C.

#### Cell viability assay

A549 and H1299 cells were seeded in 96-well plates at a density of 4000 cells per well, incubated for 24 h, and then treated with quercetin at the indicated concentrations for 48 h. The cells were then incubated with Cell Counting Kit-8 (CCK-8) solution for an additional 1 h at 37 °C and the absorbance was measured at 450 nm using a microplate reader (ThermoFisher, Waltham, MA).

#### ECM preparation

ECM powder was prepared using 95% ethanol as previously described [23]. ECM was dissolved in ethanol to make a stock solution of 200 mg/ml and then diluted in corn oil to final concentrations of 20 and 40 mg/ml.

#### In vivo animal experiments

BALB/c nude mice were obtained from Guangdong Medical Laboratory Animal Center. All mice were maintained in the specific pathogen free grade laboratory, and were subcutaneously inoculated with A549 cells (1 × 10^7^ cells mixed with 50 μl PBS and 50 μl Matrigel) into the right flank. 14 days after subcutaneous injection, the mice were randomly divided into 5 groups (seven mice per group): (1) vehicle control (solvent: 10% ethanol in normal saline); (2) ECM group (200 mg/kg ECM gavaged daily); (3) CDDP group (2 mg/kg CDDP injected i.p. once every three days); (4) CDDP + ECM-L (100 mg/kg); (5) CDDP + ECM-H (200 mg/kg). Mice were sacrificed at the end of the experiment by decapitation, and the tumors were collected for western blotting and hematoxylin and eosin (HE) staining.

### Western blotting

Samples were lysed in RIPA lysis buffer. The bicinchoninic acid assay (BCA assay) was performed to quantify protein concentrations. Equal amounts of protein were separated by SDS-PAGE using 10% polyacrylamide. Next, proteins were transferred to PVDF membranes. Membranes were blocked with 5% fat-free milk, cut into strips, and incubated with primary antibodies and secondary antibodies. Detection was performed using an enhanced chemiluminescent kit (Millipore, MA, USA). Images were acquired using a ChemiDoc XRS system with Quantity One software (Bio-Rad, Richmond, CA, USA).

### HE staining

The tumor tissue was collected and fixed in 4% paraformaldehyde. Then the samples were dehydrated with an alcohol concentration gradient and xylenes and then embedded in paraffin. For HE experiments, Sects. (4 μm) were stained with hematoxylin and eosin, and the slides were observed and photographed under a light microscope.

### Statistical analysis

Statistical analyses were performed using SPSS software 17.0 and GraphPad Prism 7.0. Data shown is from at least three independent experiments (mean ± standard error of the mean (SEM)). To determine differences, unpaired t-tests and one-way ANOVA were performed. *P*-values < 0.05 were considered statistically significant.

## Results

### Screening for *C. minima* active components and related targets

We obtained a total of 108 *C. minima* compounds from the TCMSP database. After screening for bioactive compounds using specific OB and DL conditions, we selected 21 active ingredients for further analysis, including sesquiterpenoids (plenolin, arnicolide D, arnicolide C, florilenalin angelic acid, florilenalin isobutyrate, senecioylplenolin, helenalin, 2beta-hydroxyl-2,3-dihydrogen-6-O-angeloplenolin, and 2,3,11,13-tetrahydrohelenalin), triterpenoids (taraxasteryl acetate, taraxerol, and (18alpha,19alpha)-5alpha-Urs-20(30)-ene-3beta-ol palmitate), steroids (sitosterol, stigmasterol, and beta-sitosterol), flavonoids (quercetin der., nobiletin, and quercetin), amino and amide derivatives (aurantiamide acetate), and phenols (8-hydroxy-9,10-diisobutyryloxythymol and (Z)-3,3',5,5'-Tetramethoxystilbene). Detailed information about these compounds is listed in Table 1.

**Table 1:** 21 representative components from *C. minima* and their corresponding OB, DL scores and predicted structures

Mol ID	Molecule Name	Structure	OB(%)	DL
MOL011694	Plenolin		68.26	0.19
MOL011703	2beta-hydroxyl-2,3-dihydrogen-6-O-angeloplenolin		39.36	0.4
MOL011704	(Z)-3,3',5,5'-Tetramethoxystilbene		41.69	0.21
MOL011716	8-Hydroxy-9,10-diisobutyryloxythymol		43.19	0.20
MOL011718	Arnicolide D		85.85	0.33
MOL011722	Arnicolide C		76.91	0.32
MOL011723	Florilenalin angelic acid		105.11	0.39
MOL011724	Florilenalin isobutyrate		55.53	0.38
MOL011726	Senecioylplenolin		90.22	0.37
MOL011727	(18alpha,19alpha)-5alpha-Urs-20(30)-ene-3beta-ol palmitate		33.84	0.30
MOL011728	2,3,11,13-tetrahydrohelenalin		70.36	0.19
MOL000359	Sitosterol		36.91	0.75
MOL000449	Stigmasterol		36.91	0.75
MOL004961	Quercetin der.		46.45	0.33
MOL005828	Nobiletin		61.67	0.52
MOL000596	Taraxasteryl acetate		43.08	0.74
MOL007415	Aurantiamide acetate		58.02	0.52
MOL009042	Helenalin		77.01	0.19
MOL000098	Quercetin		46.43	0.28
MOL000358	Beta-sitosterol		36.91	0.75
MOL006554	Taraxerol		38.4	0.77

### Potential target identification

**Fig. 2:**
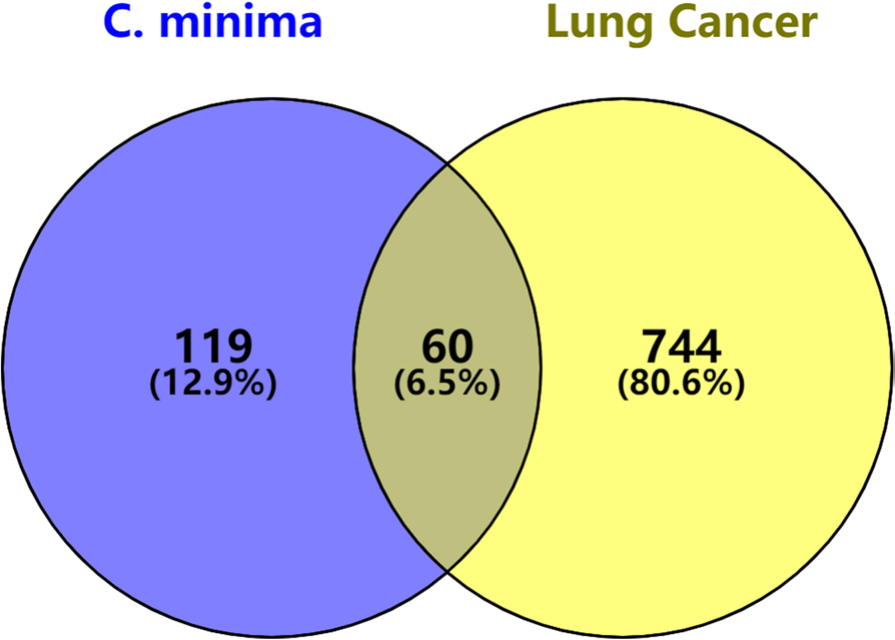
The intersection generated between *C. minima*-associated targets and lung cancer related genes

After collecting potential targets from the TCMSP database, converting them into the UniProt database, and deleting redundant items, we obtained 179 known target symbols associated with *C. minima*. We also screened a total of 804 genes related to lung cancer from the OMIM, Genecards, and Drugbank databases. We obtained a total of 60 overlapping genes (Fig. 2, see Venn diagram) and used these as *C. minima* candidate targets against lung cancer. Some of the target information is shown in Table 3.

### D-I-G-D network analyses

**Fig. 3:**
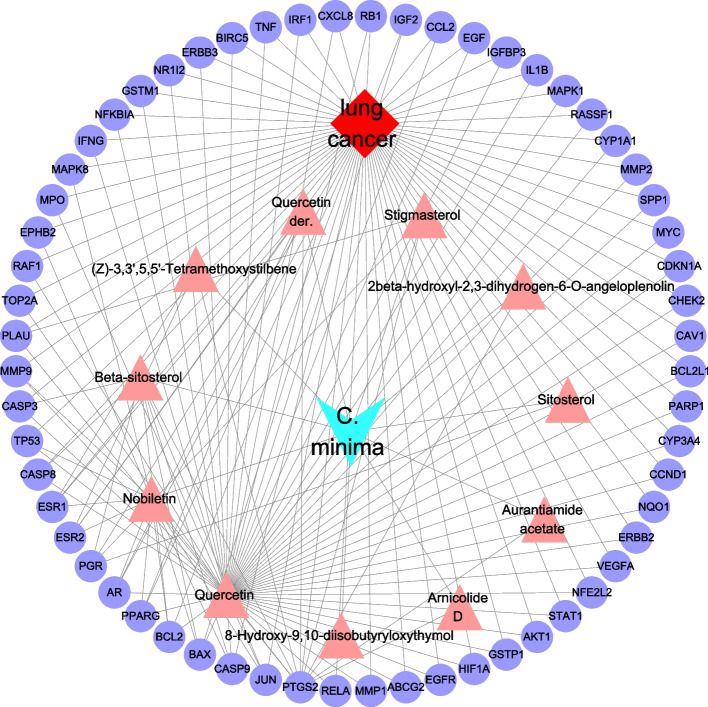
The D–I–G–D network

The D–I–G–D network contained 73 nodes (60 genes, 11 chemicals, 1 drug, and 1 disease) and 163 edges. In this network, the blue and red nodes represent *C. minima* and lung cancer, respectively. The 60 purple nodes represent the overlapping genes between *C. minima* and lung cancer, and the 11 pink nodes indicate active ingredients in *C. minima* (10 active ingredients with no common intersection targets were deleted). In addition, the degree of a node was evaluated by the proportion of edges connected to the node, which represents its importance in the network (Fig. 3). Quercetin, nobiletin and beta-sitosterol had the top three degree value, thus we considered them be the major potential *C. minima* compounds for lung cancer treatment (Table 2).

**Table 2:** Degree of 11 active components analyzed by Cytoscape

Name	Degree	Average Shortest Path Length	Betweenness Centrality	Closeness Centrality	Neighborhood Connectivity
Quercetin	56	1.29166666666666	0.4085887517578	0.774193548387096	2.625
Nobiletin	14	2.45833333333333	0.0278022162841389	0.406779661016949	4.57142857142857
Beta-sitosterol	9	2.59722222222222	0.0114861722470546	0.385026737967914	5.33333333333333
Quercetin der.	6	2.68055555555555	0.00466774653605682	0.373056994818652	6.33333333333333
(Z)-3,3',5,5'-Tetramethoxystilbene	4	2.73611111111111	0.00200896612667914	0.365482233502538	7.5
Stigmasterol	4	2.73611111111111	0.00274291885661905	0.365482233502538	7.25
2beta-hydroxyl-2,3-dihydrogen-6-O-angeloplenolin	4	2.79166666666666	0.0000734712997419672	0.35820895522388	11
8-Hydroxy-9,10-diisobutyryloxythymol	2	2.79166666666666	0.0000734712997419672	0.35820895522388	11
Arnicolide D	2	2.79166666666666	0.0000734712997419672	0.35820895522388	11
Sitosterol	2	2.79166666666666	0.000507139860644078	0.35820895522388	7.5
Aurantiamide acetate	2	2.79166666666666	0.0000734712997419672	0.35820895522388	11

### PPI network analyses

**Fig. 4:**
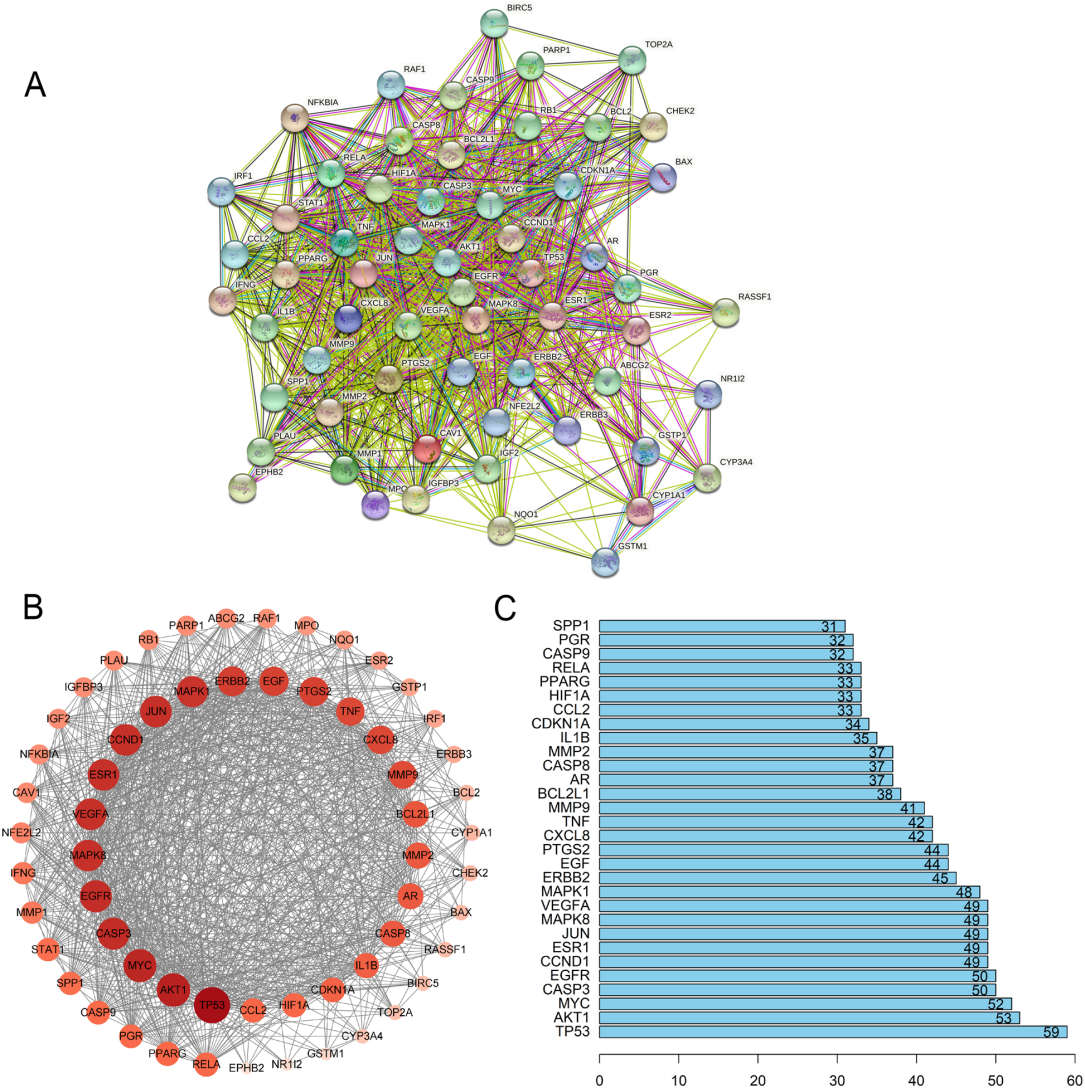
**A** The PPI network. **B** Interaction between these genes. **C** The bar plot of the PPI network

**Fig. 5:**
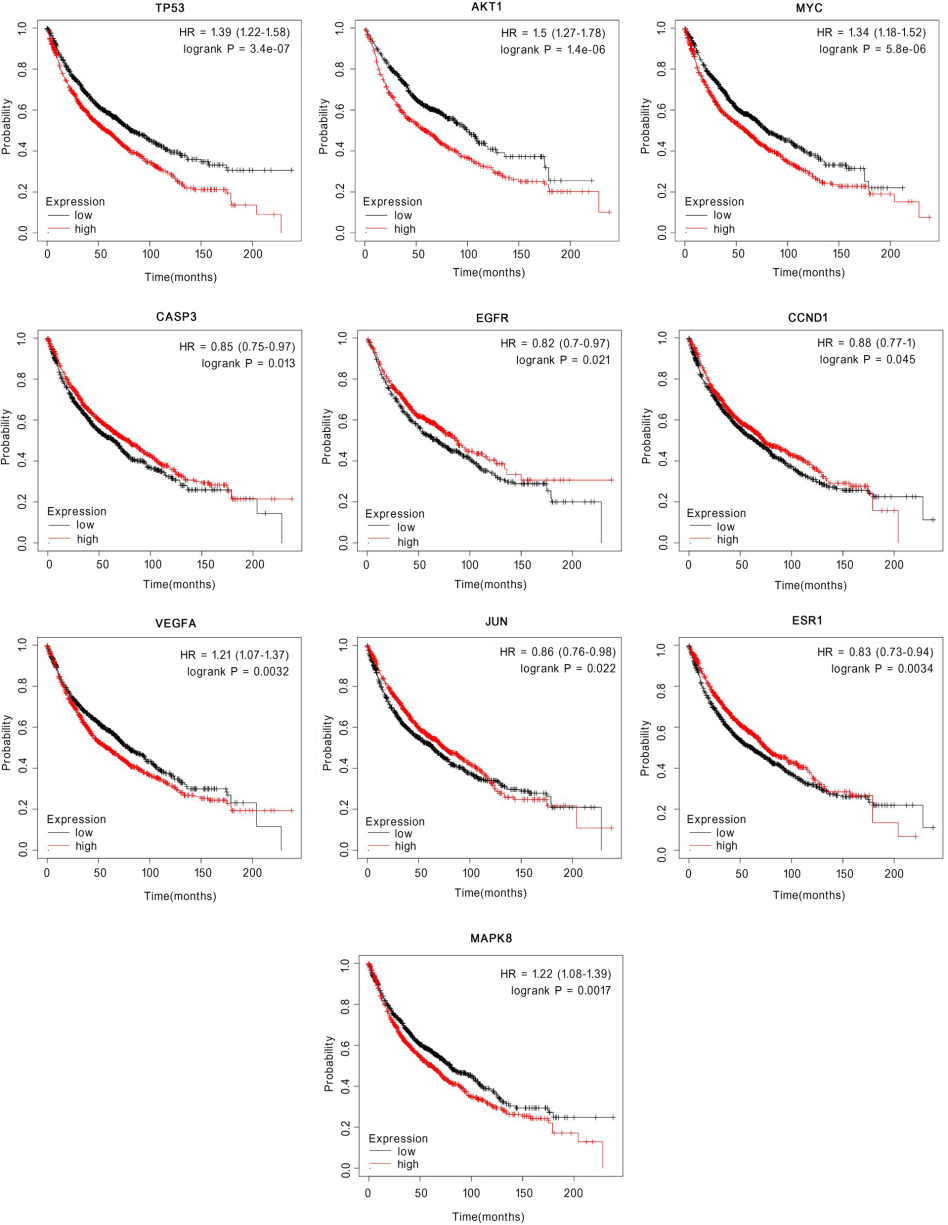
The overall survival curve of lung cancer patients with high or low expression levels of TP53, AKT1, MYC, CASP3, EGFR, CCND1, ESR1, JUN, MAPK8, and VEGFA were determined using Kaplan–Meier analysis

The constructed PPI network contained 60 nodes and 961 edges (Fig. 4A). Nodes and edges represent proteins and the connections between two proteins, respectively. We visualized the String result predictions using Cytoscape software (Fig. 4B). The node colors (light to dark) and sizes (small to large) are proportional to the node degrees. Nodes with a higher degree are considered to be more important in the network. The top 30 identified hub genes with the highest degree of connectivity were: TP53, AKT1, MYC, CASP3, EGFR, CCND1, VEGFA, JUN, ESR1, MAPK8, MAPK1, ERBB2, EGF, PTGS2, CXCL8, TNF, MMP9, BCL2L1, AR, MMP2, CASP8, IL1B, CDKN1A, PPARG, CCL2, RELA, HIF1A, PGR, CASP9, and SPP1. Among these genes, TP53 (degree = 59), AKT1 (degree = 53) and MYC (degree = 52) showed the three highest node degrees (Fig. 4C and Table 3). Survival analysis indicated that expression of top ten genes were closely linked to the lung cancer prognosis (Fig. 5), which was consistent with the results above.

**Table 3:** Degree of core regulatory genes analyzed by Cytoscape

Name	Symbol	Degree	Average Shortest PathLength	Betweenness Centrality	Closeness Centrality	Neighborhood Connectivity
Cellular tumor antigen p53	TP53	59	1	0.0722608505365032	1	30.2203389830508
RAC-alpha serine/threonine-protein kinase	AKT1	53	1.10169491525423	0.0315358277834266	0.907692307692307	32.4528301886792
Myc proto-oncogene protein	MYC	52	1.11864406779661	0.0255695601596003	0.893939393939394	32.6153846153846
Caspase-3	CASP3	50	1.15254237288135	0.0208417737179668	0.867647058823529	33.38
Epidermal growth factor receptor	EGFR	50	1.15254237288135	0.0227701783764299	0.867647058823529	33.38
G1/S-specific cyclin-D1	CCND1	49	1.16949152542372	0.021544091177951	0.855072463768116	32.938775510204
Vascular endothelial growth factor A	VEGFA	49	1.16949152542372	0.022790927250528	0.855072463768116	33.734693877551
Transcription factor AP-1	JUN	49	1.16949152542372	0.0205966593849399	0.855072463768116	33.7551020408163
Estrogen receptor	ESR1	49	1.16949152542372	0.0261671699202626	0.855072463768116	32.7551020408163
Mitogen-activated protein kinase 8	MAPK8	49	1.16949152542372	0.0181275332954268	0.855072463768116	33.7959183673469
Mitogen-activated protein kinase 1	MAPK1	48	1.1864406779661	0.0218192038686717	0.842857142857142	33.75
Receptor tyrosine-protein kinase erbB-2	ERBB2	45	1.23728813559322	0.0156141252299837	0.808219178082191	34.0666666666666
Pro-epidermal growth factor	EGF	44	1.25423728813559	0.0122804258465005	0.797297297297297	35.5681818181818
Prostaglandin G/H synthase 2	PTGS2	44	1.25423728813559	0.0125590212737923	0.797297297297297	35.4090909090909
Interleukin-8	CXCL8	42	1.28813559322033	0.0141048839673417	0.776315789473684	35.8571428571428
Tumor necrosis factor	TNF	42	1.28813559322033	0.00833145827093525	0.776315789473684	36.2380952380952
Matrix metalloproteinase-9	MMP9	41	1.30508474576271	0.00947791951541008	0.766233766233766	36.4878048780487
Bcl-2-like protein 1	BCL2L1	38	1.35593220338983	0.00798927862460732	0.7375	36.6052631578947
Androgen receptor	AR	37	1.3728813559322	0.010351431218464	0.728395061728395	36.081081081081
72 kDa type IV collagenase	MMP2	37	1.3728813559322	0.0062354852353676	0.728395061728395	38

### GO function enrichment analysis

**Fig. 6:**
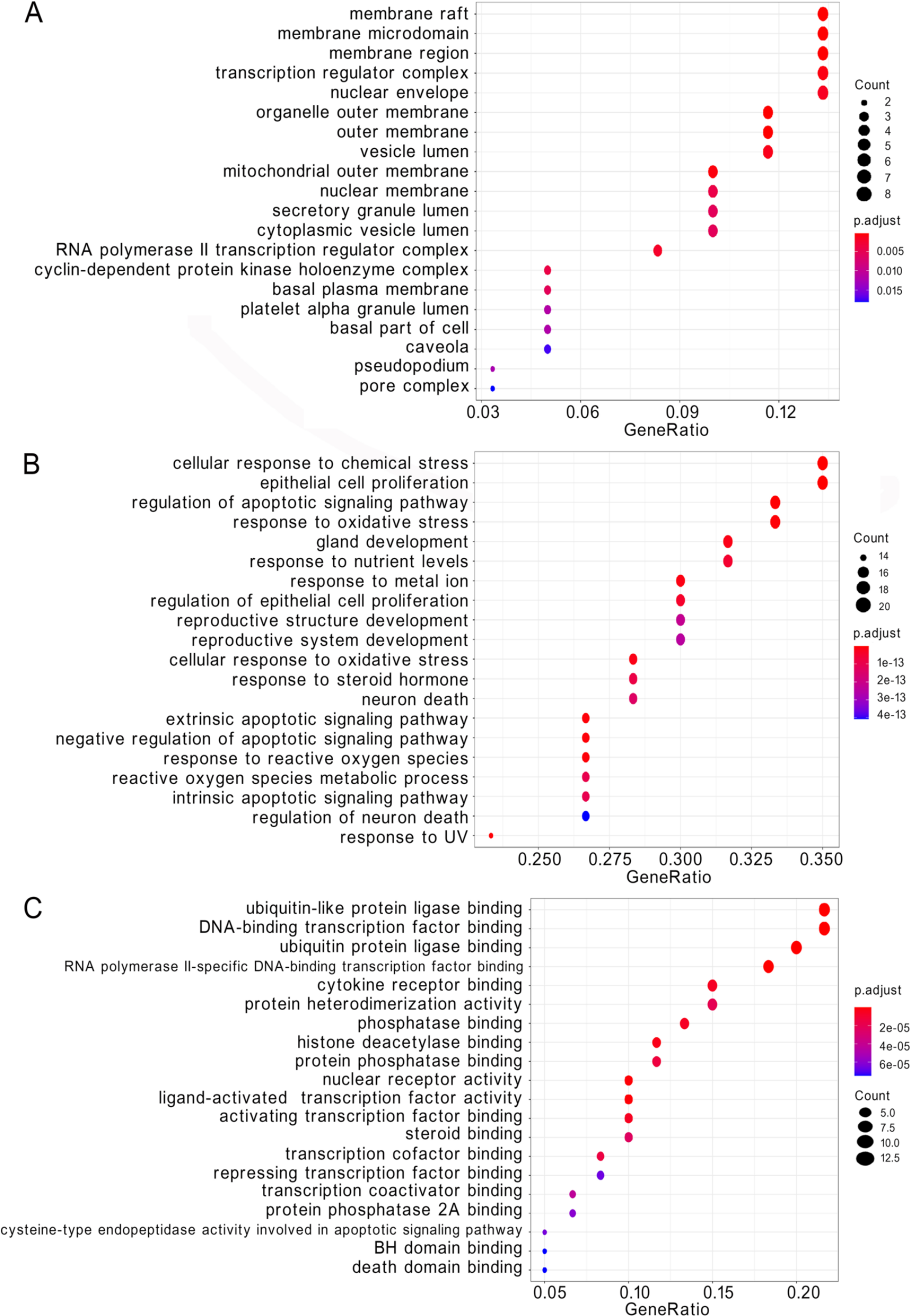
GO function enrichment analysis. **A** Cellular component category. **B** Biological process category. **C** Molecular function category

We performed three categories of GO functional annotation analysis on the 60 potential targets, and the top 20 significant terms from each category are shown in Fig. 5. Overall, we identified 1771 GO functions, including 1630 BP functions, 30 CC functions, and 111 MF functions. In the CC category, organelle outer membrane, outer membrane, membrane raft, membrane microdomain, and membrane region were enriched (Fig. 6A). In the BP category, cellular response to chemical stress, epithelial cell proliferation, regulation of apoptotic signaling pathway, response to oxidative stress, and response to UV were enriched (Fig. 6B). In the MF category, ubiquitin-like protein ligase binding, DNA-binding transcription factor binding, ubiquitin protein ligase binding, RNA polymerase II-specific DNA-binding transcription factor binding, and nuclear receptor activity were enriched (Fig. 6C).

### KEGG pathway enrichment analysis

**Fig. 7:**
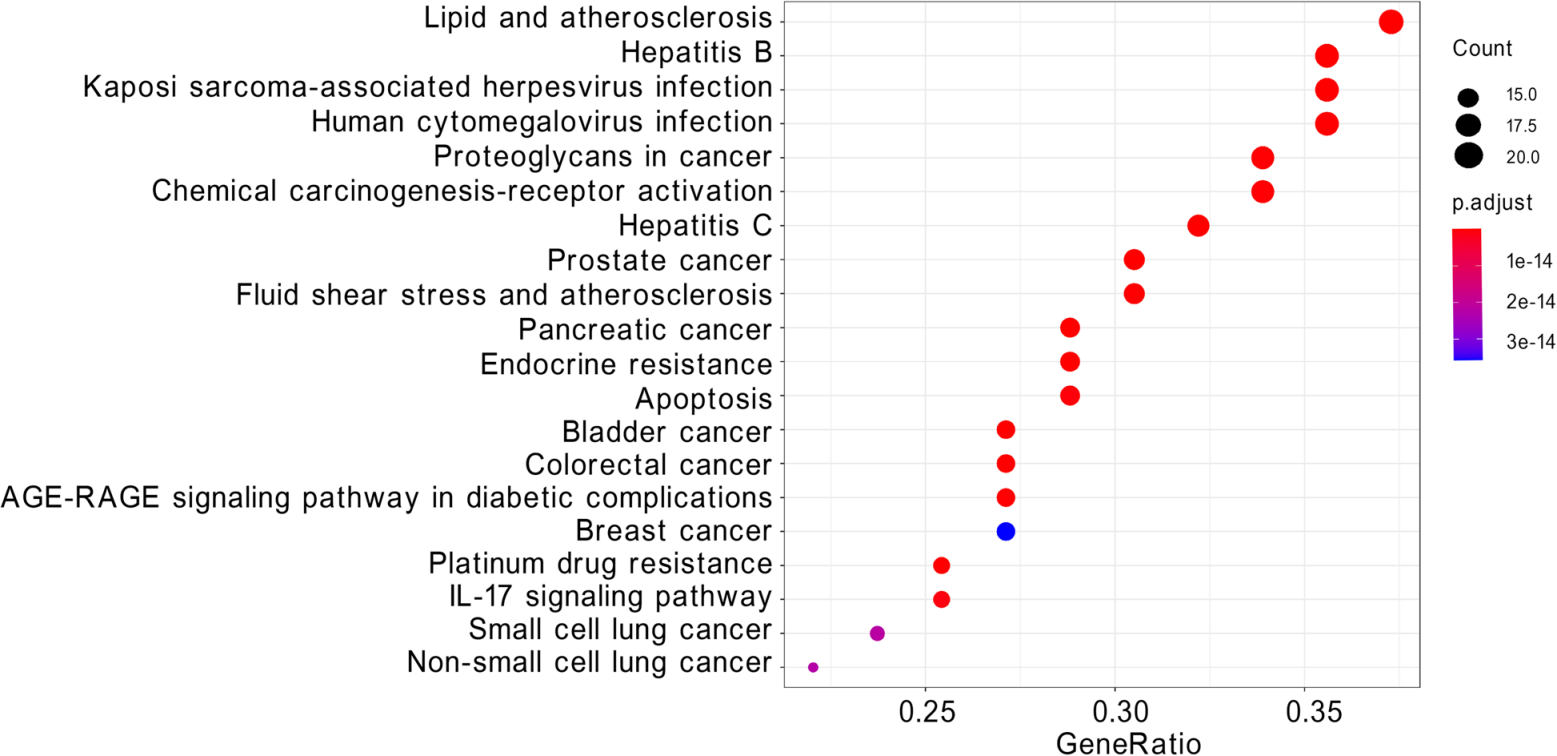
Bubble chart of KEGG pathway enrichment analysis

We identified a total of 144 pathways significantly associated with target genes. The top 20 significant pathways included cancer pathways, platinum drug resistance, apoptosis, and IL-17 signaling pathway (Fig. 7, Table 4). These pathways may be involved in the molecular mechanisms underlying *C. minima*’s impact on lung cancer.

**Table 4:** KEGG pathway enrichment analysis

ID	Description	P value	P.adjust	Q value	Count
hsa05219	Bladder cancer	6.34E-25	1.29E-22	3.74E-23	16
hsa05212	Pancreatic cancer	1.06E-21	1.07E-19	3.10E-20	17
hsa05161	Hepatitis B	1.58E-21	1.07E-19	3.10E-20	21
hsa05215	Prostate cancer	2.22E-21	1.13E-19	3.27E-20	18
hsa05417	Lipid and atherosclerosis	2.84E-20	1.16E-18	3.34E-19	22
hsa05167	Kaposi sarcoma-associated herpesvirus infection	7.56E-20	2.57E-18	7.42E-19	21
hsa01522	Endocrine resistance	1.14E-19	3.32E-18	9.58E-19	17
hsa05210	Colorectal cancer	4.74E-19	1.21E-17	3.49E-18	16
hsa05160	Hepatitis C	7.11E-19	1.61E-17	4.66E-18	19
hsa01524	Platinum drug resistance	1.38E-18	2.82E-17	8.14E-18	15
hsa05163	Human cytomegalovirus infection	1.73E-18	3.21E-17	9.27E-18	21
hsa05418	Fluid shear stress and atherosclerosis	1.99E-18	3.39E-17	9.80E-18	18
hsa05205	Proteoglycans in cancer	5.58E-18	8.75E-17	2.53E-17	20
hsa04933	AGE-RAGE signaling pathway in diabetic complications	6.14E-18	8.95E-17	2.59E-17	16
hsa05207	Chemical carcinogenesis—receptor activation	1.09E-17	1.48E-16	4.29E-17	20
hsa04210	Apoptosis	3.80E-17	4.85E-16	1.40E-16	17
hsa04657	IL-17 signaling pathway	7.92E-17	9.51E-16	2.75E-16	15
hsa05222	Small cell lung cancer	1.92E-15	2.18E-14	6.30E-15	14
hsa05223	Non-small cell lung cancer	2.09E-15	2.24E-14	6.47E-15	13
hsa05224	Breast cancer	3.50E-15	3.57E-14	1.03E-14	16

### Compound-target interaction validation by molecular docking

**Fig. 8:**
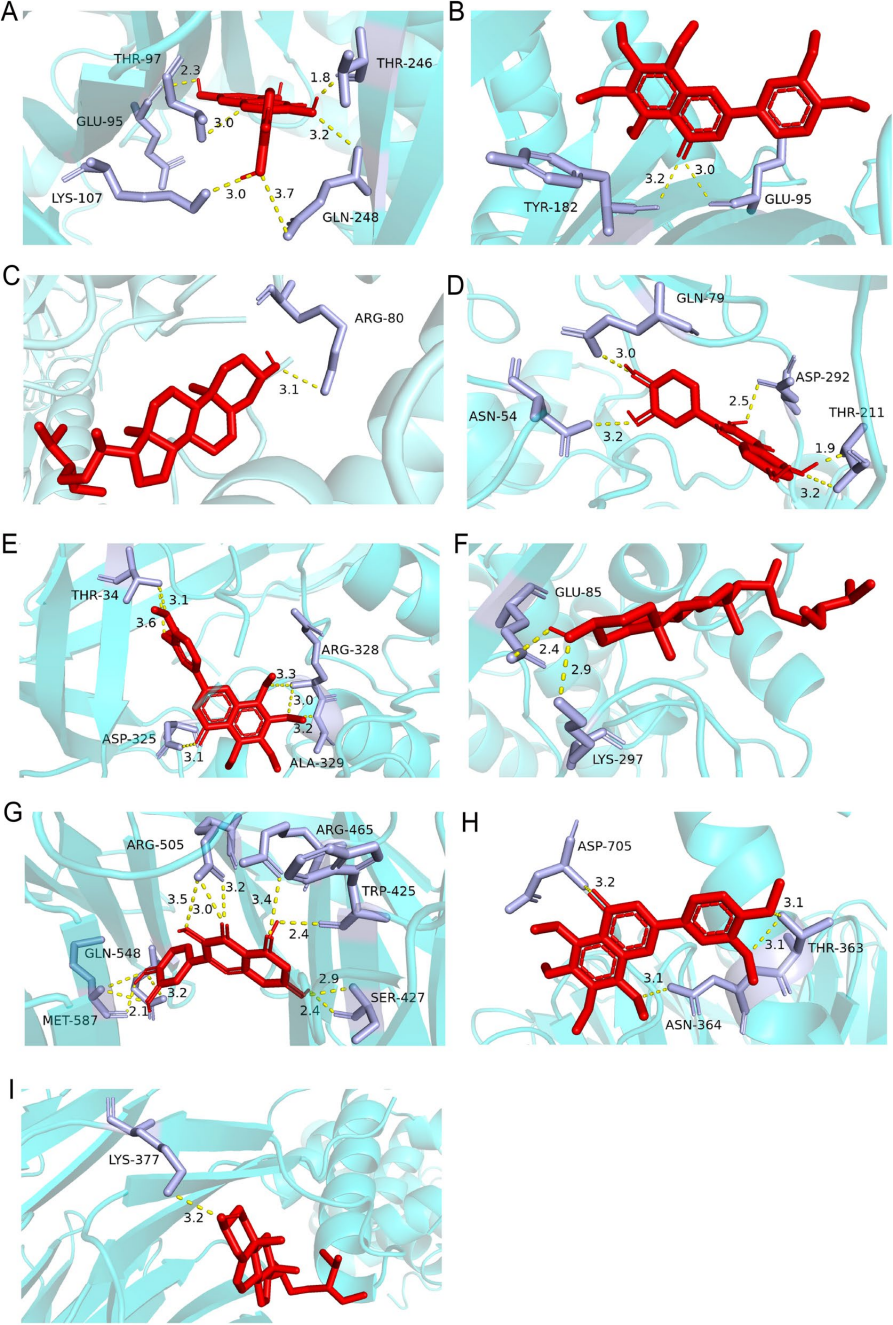
Predicted binding mode of quercetin, nobiletin, beta-sitosterol with targets obtained from AutoDock Vina. **A** Quercetin-TP53. **B** Nobiletin-TP53. **C** Beta-sitosterol-TP53. **D** Quercetin-AKT1. **E** Nobiletin-AKT1. **F** Beta-sitosterol-AKT1. **G** Quercetin-MYC. **H** Nobiletin-MYC. **I** Beta-sitosterol-MYC

To further confirm the binding capability between active drugs and important targets, we performed molecular docking. The three top targets with the highest degrees from the PPI network (TP53 (PDB ID:7BWN), AKT1 (PDB ID:4EJN), and MYC (PDB ID:7T1Z)) were considered key targets. Quercetin (PubChem CID:5,280,343), nobiletin (PubChem CID:72,344) and beta-sitosterol (PubChem CID:222,284), which had the highest degree in the active components target network, were considered major active compounds. At the same time, Erlotinib was used to be docked with the three proteins as a positive drug [24]. The details of docking results were shown in Fig. 8, and Table 5. All of the binding energy values were smaller than -5 kJ∙mol − 1, suggesting that they have good binding activity. Erlotinib was chosen as positive control, and the binding energies of quercetin, nobiletin and beta-sitosterol were lower than that of Erlotinib, indicating that the binding stability of quercetin, nobiletin and beta-sitosterol was better than that of Erlotinib. Besides, we found that quercetin shows the lowest binding affinity. Molecular docking analysis showed that quercetin mainly forms six hydrogen bonds with residues THR-97, GLU-95, LYS-107, THR-246 and GLN-248 on TP53 protein (Fig. 8A), five hydrogen bonds with residues THR-211, ASP-292, GLN-97, and ASN-54 on AKT1 protein (Fig. 8D), and eleven hydrogen bonds with residues SER-427, TRP-425, ARG-465, ARG-505, MET-587, and GLN-548 on MYC protein (Fig. 8G).

**Table 5:** The binding energy of compound and core targets (kcal/mol)

Target	Target (PDB ID)	Target Structure	Compound	Affinity (kcal/mol)
TP53	7BWN		quercetin nobiletin beta-sitosterol Erlotinib	-8.8 -7.4 -8.4 -7.3
AKT1	4EJN		quercetin nobiletin beta-sitosterol Erlotinib	-9.4 -6.7 -7.1 -6.5
MYC	7T1Z		quercetin nobiletin beta-sitosterol Erlotinib	-9.0 -6.6 -7.2 -5.9

### Quercetin induced NSCLC cell death

**Fig. 9:**
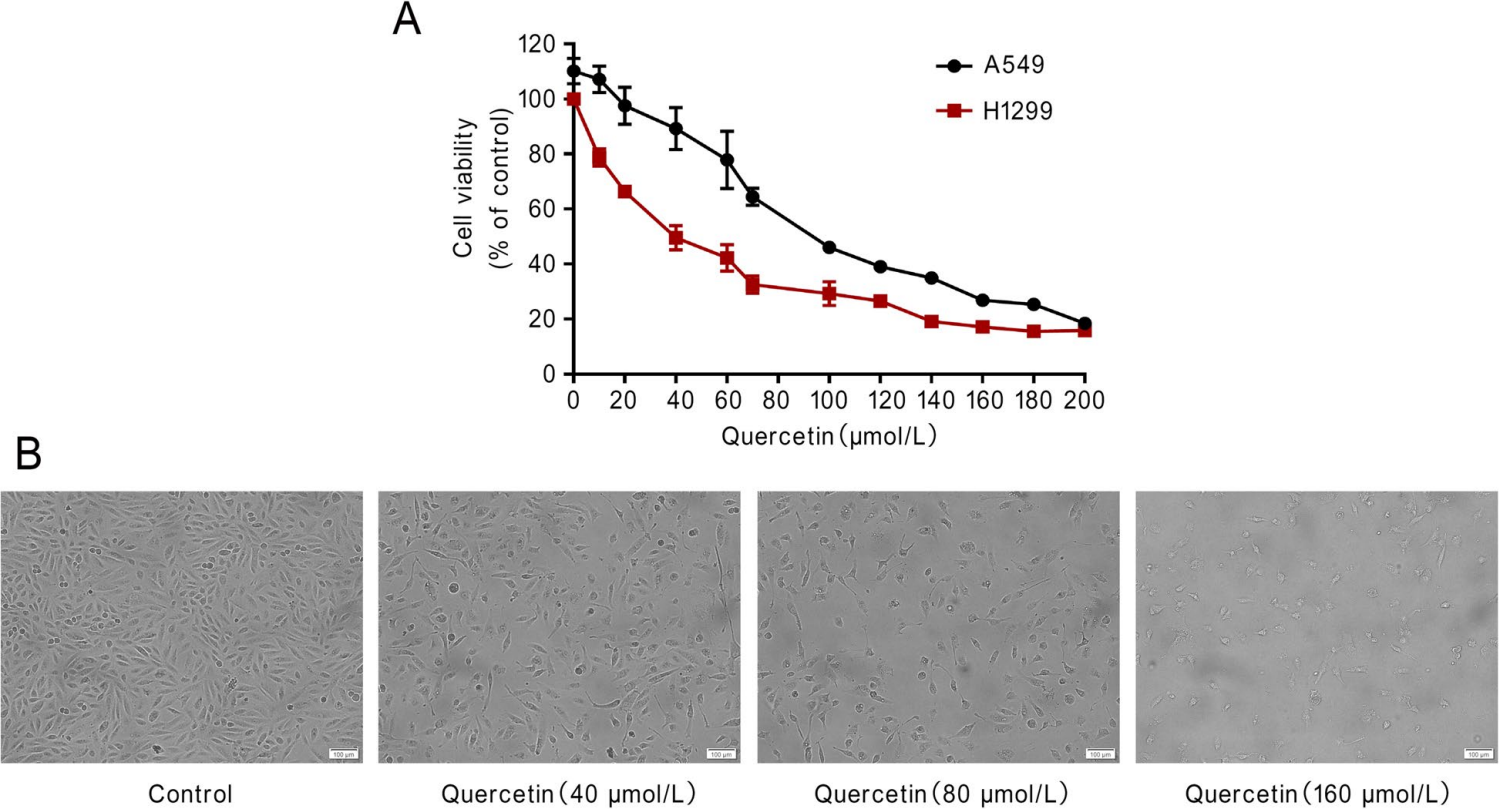
Quercetin induced cell death of NSCLC. **A** CCK8 assay was conducted in A549 and H1299 cells treated with quercetin for 48 h. **B** A549 cells were photographed and shown after being treated by quercetin for 48 h

To investigate how quercetin from *C. minima* impacts lung cancer cells, we performed a cell viability assay. We found that quercetin treatment reduced NSCLC cell viability in a dose-dependent manner (Fig. 9A). After quercetin exposure, the number of adherent cells decreased, and cells shrank and became round (Fig. 9B). These results demonstrated that quercetin had significant cytotoxic effects on NSCLC cells, which was consistent with the network pharmacology results.

### ECM enhances CDDP-induced cell apoptosis in NSCLC xenografts

**Fig. 10:**
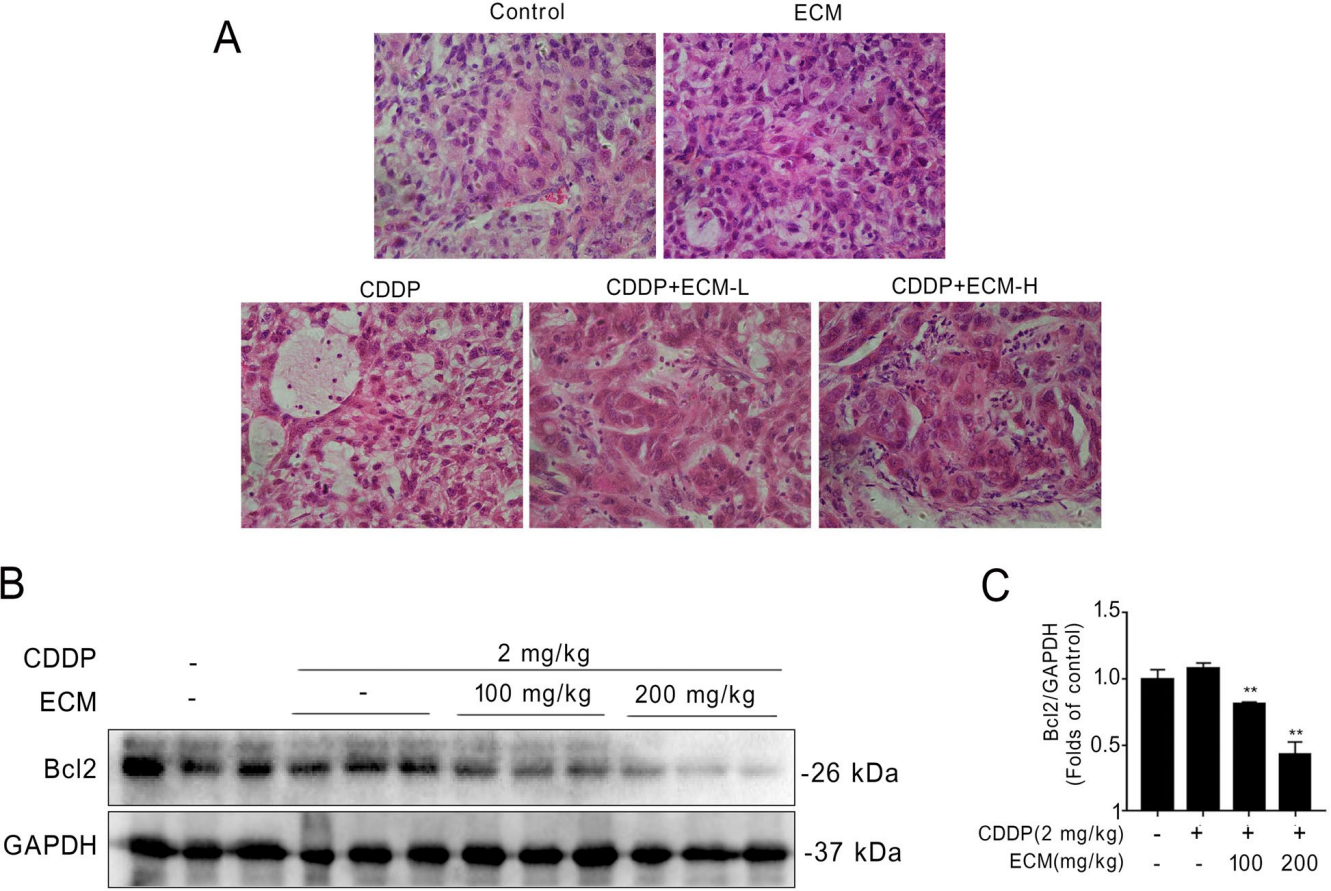
*C.minima* enhances CDDP induced cell apoptosis in NSCLC xenografts. **A** A549 cells were subcutaneously inoculated in nude mice. Representative pictures with HE staining of tumor tissues were shown. **B** Bcl2 protein levels in tumor tissues were detected by Western blotting. **C** The relative protein levels of Bcl2 were determined using densitometry analysis. ECM-L, ECM 100 mg/kg; ECM-H, ECM 200 mg/kg. ^**^ *p* < 0.01 vs. CDDP-treatment group

KEGG pathway enrichment analysis suggested that the platinum drug resistance pathway and apoptosis pathways may be involved in *C. minima*’s effects on lung cancer cells. To validate these results, we performed in vivo animal studies. CDDP as a most common platinum drug, has been widely used in the first-line treatment of lung cancer. We found that CDDP or ECM administration alone slightly induced apoptosis. However, ECM and CDDP co-treatment robustly increased the apoptosis level (Fig. 10A). Bcl2 belongs to the anti-apoptotic Bcl2 family and is required for cancer cells to survive [25]. Consistently, combined ECM and CDDP treatment reduced Bcl2 expression compared with CDDP treatment alone (Fig. 10B, C). These results demonstrated that ECM enhanced CDDP’s anti-cancer effects, at least partly, due to apoptosis pathway activation, consistent with the network pharmacology results.

## Discussion

TCM is a complementary or alternative medical system used to treat various diseases[26] and is also a potential source of candidate resources for cancer treatment. *C. minima*, a Chinese herb commonly used in clinical practice, has been shown to have anti-cancer [10, 27], anti-proliferative [15, 28], antioxidant [29], anti-inflammatory [30–32] and antibacterial activities [33, 34]. In our previous study, we confirmed the herb’s anti-cancer effects in NSCLC both in vitro and in vivo [16], but the underlying mechanism was unclear. The basic network pharmacology theory is consistent with TCM’s holistic principals and is well suited for analyzing multi-targeted Chinese medicine [35–37]. Therefore, we used a network pharmacology approach that combined screening for active components, drug targeting, network analysis, and pathway analysis to explore the therapeutic mechanisms underlying lung cancer treatment with *C. minima*.

We screened 21 potential compounds from *C. minima* and, eventually, 11 compounds were included in our D-I-G-D network, including sesquiterpenoids (arnicolide D and 2beta-hydroxyl-2,3-dihydrogen-6-O-angeloplenolin), steroids (sitosterol, stigmasterol, and beta-sitosterol), flavonoids (quercetin der., nobiletin, and quercetin), amino and amide derivatives (aurantiamide acetate), and phenols (8-hydroxy-9, 10-diisobutyryloxy-thymol, and 1-[(Z)-2-(3,5-dimethoxyphenyl)vinyl]-3,5-dimethoxybenzene). Our results indicated that these 11 compounds may be related to *C. minima*’s anti-cancer effects. According to the 11 compounds’ D-I-G-D network degrees and molecular docking analysis, quercetin emerged as a key *C. minima* active compound against lung cancer. Quercetin is a member of the flavonoid subclass of flavonols and has been found to exhibit significant anti-tumor properties [38]. Quercetin has been shown to inhibit proliferation in several cancer cell lines [39–41], and one study found that quercetin had anti-tumor activity in NSCLCs harboring wild-type EGFR by inhibiting AXL and inducing apoptosis [42]. Another study found that quercetin induced pro-apoptotic autophagy in A549 and H1299 cells [43]. Our in vitro experiment confirmed quercetin’s anti-tumor effects in NSCLC.

Further, our results suggest that TP53, AKT1, and MYC may be key targets for *C. minima* active compound anti-lung cancer activity. TP53 encodes p53 tumor suppressor protein and acts as a critical failsafe mechanism for cellular anti-cancer defenses [44–46]. TP53 has been implicated in a number of fundamental biological processes including DNA repair, cell-cycle arrest, apoptosis, autophagy, senescence, metabolism, and aging [47–50]. A TP53 tumor suppressor gene mutation occurs in approximately 50% of NSCLC cases [51]. Alpha subunit protein kinase B (AKT1), a member of the serine/threonine protein kinase family, has been shown to have a role in a variety of tumors, such as prostate cancer, osteosarcoma, ovarian cancer, and endometrial cancer, by inducing apoptosis, inhibiting tumor cell proliferation, and decreasing tumor cell invasion and metastatic capacity [52–55]. AKT1 inhibition has been reported to promote migration and invasion of NSCLC cells with KRAS or EGFR mutations in vitro [56]. Wu et al. found that miR-377-5p can inhibit lung cancer cell proliferation, invasion, and cell cycle progression by targeting AKT1 signaling [57]. Thus, AKT1 has emerged as an attractive target for new anti-cancer therapy development. MYC consists of 3 paralogs, C-MYC, N-MYC, and L-MYC, that are frequently deregulated driver genes in human cancers [58]. A previous study found that MYC-induced long non-coding RNAs inhibited NSCLC cell cycle arrest and apoptosis by activating C-MYC and its downstream effectors [59]. In our study, we used AutoDock Vina and PyMOL software to investigate quercetin’s possible binding sites with related proteins and found that quercetin had strong binding activity with TP53, AKT1, and MYC.

CDDP has been widely used in first-line lung cancer treatment [60]. However, the development of chemoresistance remains a major obstacle for treatment success [61]. We used GO enrichment analysis to annotate protein target functions in three main categories: BP, CC, and MF. Our KEGG pathway analysis revealed that these targets were significantly enriched in a variety of cancer pathways, platinum drug resistance, and apoptosis pathways, interesting areas for further research. Notably, recent research found that quercetin could overcome CDDP resistance in cervical cancer cells [62]. Additionally, our previous research found that ECM can sensitize NSCLC cells to CDDP and hypersensitize CDDP-induced apoptosis in NSCLC, but the underlying mechanism was unclear [16]. In the present study, western blotting and HE staining showed that ECM could significantly enhance CDDP-induced apoptosis. All of these experimental results validated our network pharmacology prediction results.

Based on our network pharmacology results and experimental evidence from previous work, *C. minima* clearly has the potential for use in developing new NSCLC treatments. Additionally, our study demonstrated that network pharmacology predictions can be used to characterize TCM pharmacological mechanisms in detail. Evidence suggests that *C. minima* functions as an anti-cancer drug through multiple targets, amongst which inducing apoptosis and influencing platinum drug resistance were pathways identified by both in vivo and in vitro experiments. However, the current work has several limitations. First, more comprehensive TCM target gene databases are needed to obtain more reliable results. Furthermore, it is necessary to explore how the three hub genes change in response to single-drug therapy or two-drug combination therapy. Finally, although we determined that quercetin was the most important bioactive compound in *C. minima*, it may not be completely interchangeable for *C. minima*. Thus, further studies investigating *C. minima*’s anti-lung cancer molecular mechanisms are needed.

## Conclusions

Here we used network pharmacology along with molecular docking and experimental validation to explore *C. minima*’s anti-lung cancer material basis and mechanism of action. We found that quercetin was the key active ingredient and that TP53, AKT1, and MYC may be three potential *C. minima* targets in lung cancer treatment. Additionally, we found that *C. minima* may exert anti-cancer effects by inducing apoptosis and influencing platinum drug resistance. In conclusion, our research provides a reference for further study of *C. minima*’s potential clinical application for lung cancer treatment.

### Supplementary Information

**Additional file 1.** Western blotting of GAPDH. Western blotting of Bcl2.

## Data Availability

The datasets used and/or analyzed in the current study are available from the corresponding author upon reasonable request.

## Abbreviations

*C. minima*: Centipeda minima (L.)A.Br.et Aschers
NSCLC: Non-small cell lung cancer
ECM: Ethanol extract of *C. minima*
TCM: Traditional Chinese medicine
CDDP: Cisplatin
TCMSP: Traditional Chinese Medicine Systems Pharmacy database and analysis platform
OMIM: Online Mendelian Inheritance in Man
D-I-G-D: Drug-ingredients-gene symbols-disease
PPI: Protein–protein interaction
GO: Gene Ontology
KEGG: Kyoto Encyclopedia of Genes and Genomes
TP53: Cellular tumor antigen p53
AKT1: RAC-alpha serine/threonine-protein kinase
MYC: V-myc myelocytomatosis viral oncogene homolog
OB: Oral bioavailability
DL: Drug-likeness
CC: Cellular component
BP: Biological process
MF: Molecular function

## References

[1] Sung H (2021). Global cancer statistics 2020: GLOBOCAN estimates of incidence and mortality worldwide for 36 cancers in 185 countries. Ca-a Cancer J Clin.

[2] Bade BC, Dela Cruz CS (2020). Lung cancer 2020 epidemiology, etiology, and prevention. Clin Chest Med.

[3] Jones GS, Baldwin DR (2018). Recent advances in the management of lung cancer. Clin Med.

[4] Herbst RS, Morgensztern D, Boshoff C (2018). The biology and management of non-small cell lung cancer. Nature.

[5] Alexander M, Kim SY, Cheng HY (2020). Update 2020: management of non-small cell lung cancer. Lung.

[6] Zang FR (2020). Shikonin suppresses NEAT1 and Akt signaling in treating paclitaxel-resistant non-small cell of lung cancer. Mole Med.

[7] Efferth T (2007). From traditional Chinese medicine to rational cancer therapy. Trends Mole Med.

[8] Yan ZK, Lai ZJ, Lin JM (2017). Anticancer properties of traditional Chinese medicine. Combinatorial Chem High Throughput Screen.

[9] Guan YM, Zhang NS, Zhang YY (2016). First report of fusarium commune causing root and basal stem rot on centipeda minima in China. Plant Dis.

[10] Linh NT (2021). Medicinal plant centipeda minima: a resource of bioactive compounds. Mini-Rev Med Chem.

[11] Tan JC (2022). Centipeda minima: an update on its phytochemistry, pharmacology and safety. J Ethnopharmacol.

[12] Wang Y (2017). Inhibition of Nrf2 enhances the anticancer effect of 6-O-angeloylenolin in lung adenocarcinoma. Biochem Pharmacol.

[13] Liu R (2019). Brevilin A induces cell cycle arrest and apoptosis in nasopharyngeal carcinoma. Front Pharmacol.

[14] Lee MML (2020). Anti-cancer activity of centipeda minima extract in triple negative breast cancer via Inhibition of AKT, NF-kappa B, and STAT3 signaling pathways. Front Oncol.

[15] You PT (2018). Brevilin A induces apoptosis and autophagy of colon adenocarcinoma cell CT26 via mitochondrial pathway and PI3K/AKT/mTOR inactivation. Biomed Pharmacother.

[16] Fan XZ (2021). Centipeda minima extract sensitizes lung cancer cells to DNA-crosslinking agents via targeting Fanconi anemia pathway. Phytomedicine.

[17] Saikia S, Bordoloi M (2019). Molecular docking: challenges, advances and its use in drug discovery perspective. Curr Drug Targets.

[18] Shannon P (2003). Cytoscape: a software environment for integrated models of biomolecular interaction networks. Genome Res.

[19] Hsia CW (2015). Analysis of dermal papilla cell interactome using STRING database to profile the ex vivo hair growth inhibition effect of a Vinca alkaloid drug, colchicine. Int J Mol Sci.

[20] Kanehisa M (2023). KEGG for taxonomy-based analysis of pathways and genomes. Nucleic Acids Res.

[21] Chen GL, Seukep AJ, Guo MQ (2020). Recent advances in molecular docking for the research and discovery of potential marine drugs. Mar Drugs.

[22] Gaillard T (2018). Evaluation of autodock and autoDock Vina on the CASF-2013 benchmark. J Chem Inform Model.

[23] Wang YJ (2019). Ethanol extract of centipeda minima exerts antioxidant and neuroprotective effects via activation of the Nrf2 signaling pathway. Oxid Med Cell Longev.

[24] Midha A, Dearden S, McCormack R (2015). EGFR mutation incidence in non-small-cell lung cancer of adenocarcinoma histology: a systematic review and global map by ethnicity (mutMapII). Am J Cancer Res.

[25] Vogler M (2014). Targeting BCL2-proteins for the treatment of solid tumours. Adv Med.

[26] Zhou W (2016). Systems pharmacology exploration of botanic drug pairs reveals the mechanism for treating different diseases. Sci Rep.

[27] Liu R (2019). Arnicolide D, from the herb centipeda minima, is a therapeutic candidate against nasopharyngeal carcinoma. Molecules.

[28] Su MX (2010). Antiproliferative effects of volatile oils from centipeda minima on human nasopharyngeal cancer CNE cells. Nat Prod Commun.

[29] Huang SS (2013). Antioxidant and anti-inflammatory activities of aqueous extract of Centipeda minima. J Ethnopharmacol.

[30] Qin Q (2021). Brevilin A inhibits NLRP3 inflammasome activation in vivo and in vitro by acting on the upstream of NLRP3-induced ASC oligomerization. Mole Immunol.

[31] Xue PH (2021). Cytotoxic and anti-inflammatory sesquiterpenes from the whole plants of centipeda minima. J Nat Prod.

[32] Chan BD (2021). Centipeda minima extract attenuates dextran sodium sulfate-induced acute colitis in mice by inhibiting macrophage activation and monocyte chemotaxis. Front Pharmacol.

[33] Liang HX (2007). Antibacterial thymol derivatives isolated from Centipeda minima. Molecules.

[34] Taylor RS, et al. Antibacterial constituents of the Nepalese medicinal herb, Centipeda minima. Phytochemistry. 1998;47(4):631–4. 10.1016/s0031-9422(97)00534-7 9461679

[35] Chen Y (2018). Anti-endometriosis mechanism of Jiawei Foshou San based on network pharmacology. Front Pharmacol.

[36] He SB (2019). A computational toxicology approach to screen the hepatotoxic ingredients in traditional Chinese medicines: polygonum multiflorum thunb as a case study. Biomolecules.

[37] He SS (2020). Uncovering the molecular mechanism of the Qiang-Xin 1 formula on sepsis-induced cardiac dysfunction based on systems pharmacology. Oxid Med Cell Longev.

[38] Kashyap D (2019). Fisetin and Quercetin: promising flavonoids with chemopreventive potential. Biomolecules.

[39] Jeong JH (2009). Effects of low dose quercetin: cancer cell-specific inhibition of cell cycle progression. J Cell Biochem.

[40] Ren MX (2015). Effect of quercetin on the proliferation of the human ovarian cancer cell line SKOV-3 in vitro. Exp Ther Med.

[41] Granato M (2017). Quercetin induces apoptosis and autophagy in primary effusion lymphoma cells by inhibiting PI3K/AKT/mTOR and STAT3 signaling pathways. J Nutr Biochem.

[42] Huang KY (2021). Growth suppression in lung cancer cells harboring EGFR-C797S mutation by quercetin. Biomolecules.

[43] Guo HJ (2021). Quercetin induces pro-apoptotic autophagy via SIRT1/AMPK signaling pathway in human lung cancer cell lines A549 and H1299 in vitro. Thoracic Cancer.

[44] Donehower LA (2019). Integrated analysis of TP53 gene and pathway alterations in the cancer genome atlas. Cell Rep.

[45] Garancher A (2019). Tumor necrosis factor overcomes immune evasion in P53-mutant medulloblastoma. Neuro Oncol.

[46] Kastenhuber ER, Lowe SW (2017). Putting p53 in context. Cell.

[47] Cavic M (2019). TP53 and DNA-repair gene polymorphisms genotyping as a low-cost lung adenocarcinoma screening tool. J Clin Pathol.

[48] Hu W, et al. TP53, TP53 Target Genes (DRAM, TIGAR), and Autophagy. Adv Exp Med Biol. 2019;1206:127–49. 10.1007/978-981-15-0602-4_6 31776983

[49] Lacroix M (2020). Metabolic functions of the tumor suppressor p53: Implications in normal physiology, metabolic disorders, and cancer. Mole Metab.

[50] Mijit M (2020). Role of p53 in the regulation of cellular senescence. Biomolecules.

[51] Hu B (2020). High expression of CARM1 inhibits lung cancer progression by targeting TP53 by regulating CTNNB1. Lung.

[52] Alwhaibi A (2019). Nodal pathway activation due to Akt1 suppression is a molecular switch for prostate cancer cell epithelial-to-mesenchymal transition and metastasis. Biochem Pharmacol.

[53] Huo X (2019). Clinical and expression significance of AKT1 by co-expression network analysis in endometrial cancer. Front Oncol.

[54] Wang JJ (2019). MAT1 facilitates the lung metastasis of osteosarcoma through upregulation of AKT1 expression. Life Sci.

[55] Tian XY (2022). Hexokinase 2 promoted cell motility and proliferation by activating Akt1/p-Akt1 in human ovarian cancer cells. J Ovarian Res.

[56] Rao GH (2017). Inhibition of AKT1 signaling promotes invasion and metastasis of non-small cell lung cancer cells with K-RAS or EGFR mutations. Sci Rep.

[57] Wu H (2019). miR-377-5p inhibits lung cancer cell proliferation, invasion, and cell cycle progression by targeting AKT1 signaling. J Cell Biochem.

[58] Duffy MJ (2021). MYC as a target for cancer treatment. Cancer Treat Rev.

[59] Chen SJ (2019). Roles of MYC-targeting long non-coding RNA MINCR in cell cycle regulation and apoptosis in non-small cell lung cancer. Respir Res.

[60] Dho SH (2018). Development of a radionuclide-labeled monoclonal anti-CD55 antibody with theranostic potential in pleural metastatic lung cancer. Sci Rep.

[61] Wakelee H, et al. 50 Years of progress in the systemic therapy of non-small cell lung cancer. Am Soc Clin Oncol Educ Book. 2014;34:177–89. 10.14694/EdBook_AM.2014.34.177 PMC5600272 24857075

[62] Ji HH (2022). Prediction of the mechanisms by which quercetin enhances cisplatin action in cervical cancer: a network pharmacology study and experimental validation. Front Oncol.

