# Correction: Centipeda minima active components and mechanisms in lung cancer

**DOI:** 10.1186/s12906-024-04498-y

**Published:** 2024-05-15

**Authors:** Cuiyun Gao, Huafeng Pan, Fengjun Ma, Ze Zhang, Zedan Zhao, Jialing Song, Wei Li, Xiangzhen Fan

**Affiliations:** 1 Department of Rehabilitation Medicine, Binzhou Medical University Hospital, Binzhou, Shandong China; 2 School of Rehabilitation Medicine, Binzhou Medical University, Yantai, Shandong China; 3 Science and Technology Innovation Center, Guangzhou University of Chinese Medicine, Guangzhou, Guangdong China; 4 Shandong University of Traditional Chinese Medicine, Jinan, Shandong China

**Correction: BMC Complement Med Ther (2023) 23:89**

10.1186/s12906-023-03915-y

Following publication of the original article [1], the authors reported an error in in the grant number for the first fund provided in the paper.

The correct grant number should be “No. ZR2022QH001”, instead of “No. ZR202111290609”.

The original article has been corrected.
