# Supplementary figures and images for: Centipeda minima active components and mechanisms in lung cancer
Source: BMC Complement Med Ther. 2023 Mar 23;23:89. doi: 10.1186/s12906-023-03915-y (PMC10035269; doi:10.1186/s12906-023-03915-y)

**Western blotting of GAPDH**

| 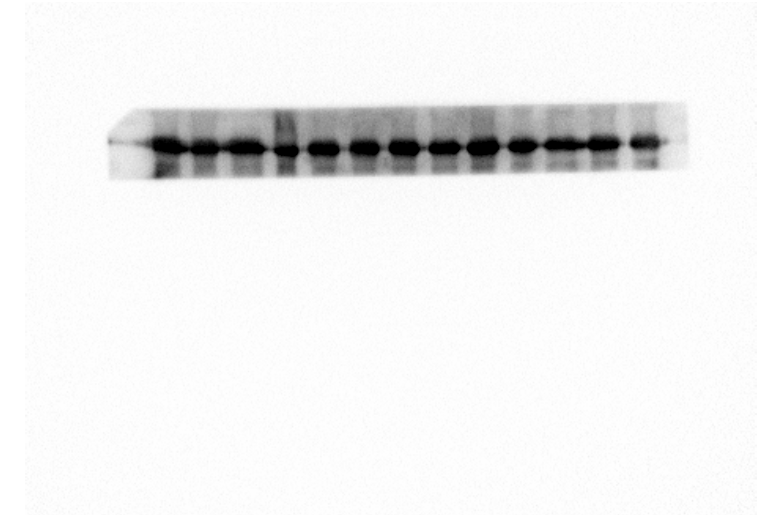 | 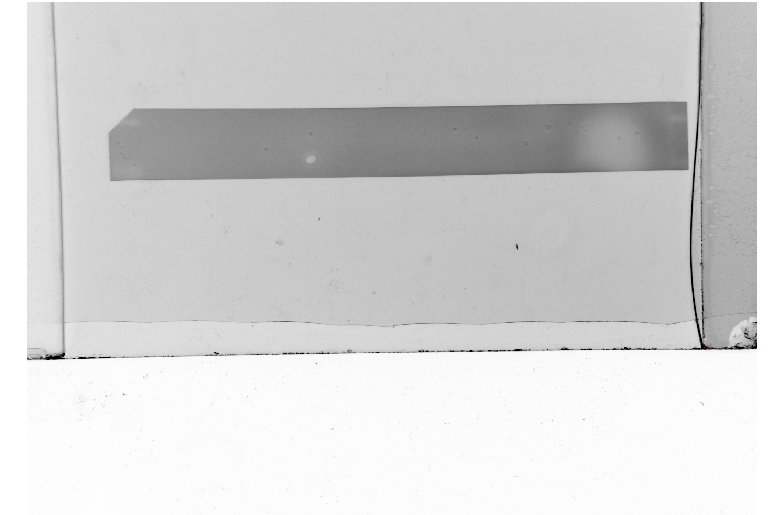 |
| --- | --- |

**Western blotting of Bcl2**

| 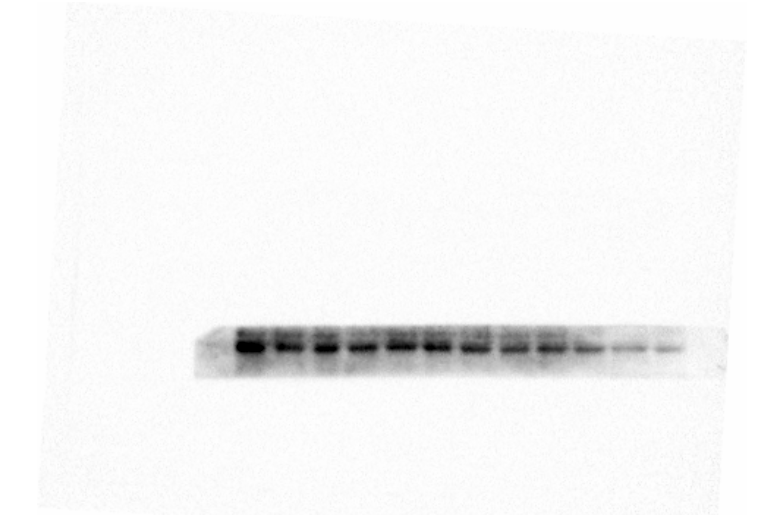 | 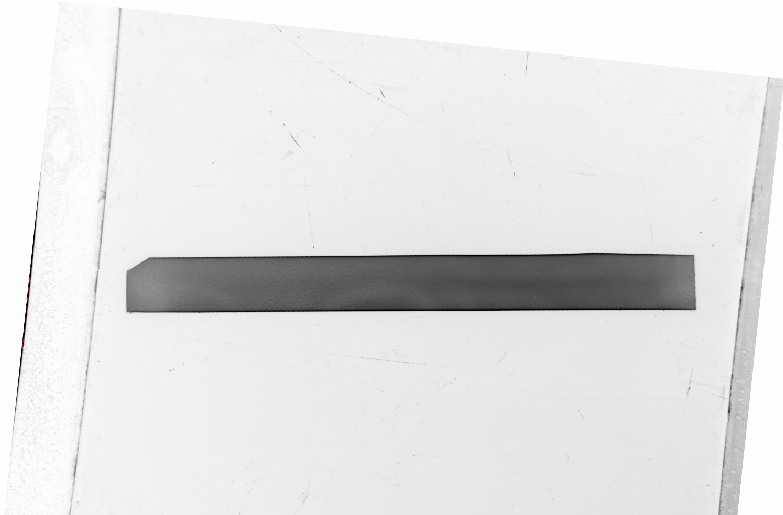 |
| --- | --- |

Supplement: Supplementary file 1 — Additional file 1. Western blotting of GAPDH. Western blotting of Bcl2. [file 12906_2023_3915_MOESM1_ESM.docx]
